# Clinical evaluation of a new rapid immunochromatographic test for detection of *Bordetella pertussis* antigen

**DOI:** 10.1038/s41598-022-11933-y

**Published:** 2022-05-16

**Authors:** Kenji Okada, Yuho Horikoshi, Naoko Nishimura, Shigeki Ishii, Hiroko Nogami, Chikako Motomura, Isao Miyairi, Naoki Tsumura, Toshihiko Mori, Kenta Ito, Shinichi Honma, Kensuke Nagai, Hiroshi Tanaka, Toru Hayakawa, Chiharu Abe, Kazunobu Ouchi

**Affiliations:** 1 Division of Basic Nursing, Fukuoka Nursing College, Fukuoka, Japan; 2 Division of Infectious Diseases, Department of Pediatrics, Tokyo Metropolitan Children’s Medical Center, Tokyo, Japan; 3 Department of Pediatrics, Konan Kosei Hospital, Aichi, Japan; 4 Department of Pediatrics, Miyazaki Prefectural Miyazaki Hospital, Miyazaki, Japan; 5 Department of Respiratory Medicine, National Hospital Organization Fukuoka National Hospital, Fukuoka, Japan; 6 Department of Pediatrics, National Hospital Organization Fukuoka National Hospital, Fukuoka, Japan; 7 Division of Infectious Diseases, Department of Medical Subspecialties, National Center for Child Health and Development, Tokyo, Japan; 8 Tsumura Family Clinic, Fukuoka, Japan; 9 Department of Pediatrics, NTT East Sapporo Hospital, Hokkaido, Japan; 10 Department of General Pediatrics, Aichi Children’s Health and Medical Center, Aichi, Japan; 11 Honma Children’s Clinic, Fukuoka, Japan; 12 Nagai Pediatric Clinic, Fukuoka, Japan; 13 Sapporo Cough Asthma and Allergy Center, Hokkaido, Japan; 14 Diagnostics Department, Asahi Kasei Pharma Corporation, 1-1-2 Yurakucho, Chiyoda-ku, Tokyo, 100-0006 Japan; 15 Department of Medical Welfare for Children, Kawasaki University of Medical Welfare, Okayama, Japan

**Keywords:** Infectious-disease diagnostics, Bacterial infection, Laboratory techniques and procedures

## Abstract

A more rapid and less complicated test to diagnose pertussis is required in clinical settings. We need to detect *Bordetella pertussis*, which mainly causes pertussis, as early as possible, because pertussis is more likely to become severe in infants, and people around them can easily become a source of infection due to its strong infectivity. Nevertheless, methods that can detect *B. pertussis* rapidly and efficiently are lacking. Therefore, we developed a new immunochromatographic antigen kit (ICkit) for the early diagnosis of pertussis. The ICkit detects *B. pertussis* antigens in a nasopharyngeal swab without equipment and provides the result in about 15 min with a simple procedure. Additionally, a prospective study to evaluate the ICkit was conducted in 11 medical institutions, involving 195 cases with suspected pertussis. Compared with the real-time polymerase chain reaction (rPCR), the sensitivity and specificity of the ICkit were 86.4% (19/22) and 97.1% (168/173), respectively. The ICkit detected the antigen in both children and adults. Furthermore, the ICkit detected the antigen until the 25th day from the onset of cough, when rPCR detected the antigen. Thus, the ICkit demonstrated a high correlation with rPCR and would help diagnose pertussis more rapidly and efficiently.

## Introduction

Pertussis, known as whooping cough, is a highly contagious respiratory infection caused mainly by *Bordetella pertussis* which spreads easily from person to person due to its strong infectivity^1^. Cases of pertussis are still frequently reported worldwide^2^.

When *B. pertussis* infects unvaccinated infants, the disease can be severe and may result in death^3,4^. Globally, it was estimated that there were about 24.1 million infected cases and 160,700 deaths due to pertussis in children under the age of five years in 2014^5^. In addition, the presence of infected adults around unvaccinated infants poses a risk of transmission to the infants^6,7^. Although pertussis is a vaccine-preventable disease, it has recently been reported that infection spreads among adults whose vaccine efficacy has diminished over time^8^.

Pertussis has non-specific symptoms such as rhinorrhea, sneezing, and non-specific cough during the catarrhal phase, and characteristic symptoms such as paroxysms, whooping, post-tussive vomiting, and apnea during the paroxysmal phase^9,10^. The severity and characteristics of the symptoms vary depending on the age of the patient, duration of infection, and vaccination^11,12^. Since there are often no specific clinical symptoms, it is difficult to diagnose based on clinical symptoms alone^13^. Adenoviruses, respiratory syncytial viruses (RSVs), human parainfluenza viruses, influenza viruses, *Haemophilus influenzae*, *Mycoplasma pneumoniae*, and other agents may also cause pertussis-like symptoms^14–16^. Infants hospitalized with acute respiratory tract infections may have pertussis without typical symptoms^17,18^. Thus, diagnosis based on laboratory confirmation is essential, along with clinical symptoms.

There are cultures, nucleic acid tests, and serological tests for the diagnosis of pertussis^9,19^. Of these tests, the direct tests such as cultures and nucleic acid tests help determine infectivity and the need for antimicrobials, unlike the indirect tests such as antibody tests. However, the existing direct tests are often time-consuming and limited in use due to facility conditions, so that the requirements for the direct tests have not yet been fully met. Culture is the gold standard for diagnosis in the World Health Organization laboratory manual, but it has low sensitivity. Additionally, it takes at least one week to obtain the results, and a specialized medium, facility, and expertise are needed. Instead of culture, nucleic acid tests are often used in clinical practice. The nucleic acid test has excellent sensitivity, but it cannot provide rapid results because it takes several hours to obtain the result. It also requires the preparation of a measurement environment that involves specialized equipment and trained personnel. Therefore, we often have difficulties getting the test results at the patient’s first visit. Especially in the clinic that patients visit initially, it is challenging to perform the tests due to a shortage of equipment and technical personnel. Thus, a novel direct test is required to enable rapid and straightforward detection without equipment.

**Figure 1:**
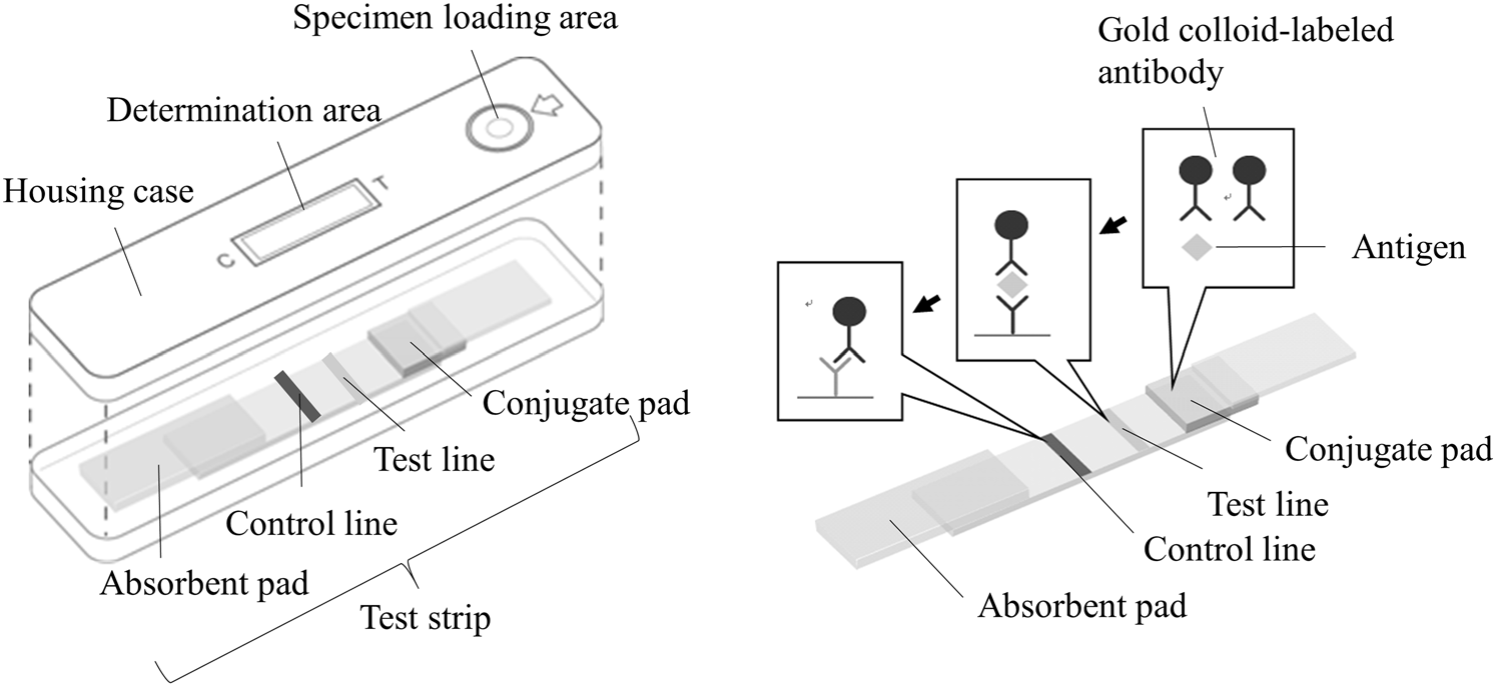
Structure and principle of the immunochromatographic antigen kit (ICkit). This product is a kit that detects *Bordetella pertussis* antigen in a nasopharyngeal swab specimen by immunochromatography. The kit includes reagent A, reagent B, and a test plate that consists of a test strip and a housing case. The specimen is applied to the test plate after pretreatment with reagent A and reagent B. The specimen moves over the test strip by capillary action. When the specimen reaches the conjugate pad, the antigen causes an antigen–antibody reaction with the gold colloid-labeled monoclonal antibody (mouse) bound in the conjugate pad and forms an immunocomplex. This immunocomplex flows to the absorbent pad through the nitrocellulose membrane by capillary action. When reaching the test line, it is captured by the immobilized monoclonal antibody to form a sandwich immunocomplex. The sandwich immunocomplex results in a purplish-red line appearing at the determination part on the test plate. The presence or absence of this line can thus determine the presence or absence of the *B. pertussis* antigen. The control line appears as a purplish-red line of gold colloid-labeled monoclonal antibody when this antibody flows over the membrane and binds to a solid-phase anti-mouse immunoglobulin polyclonal antibody (rabbit), irrespective of the presence or absence of the *B. pertussis* antigen.

A new rapid immunochromatographic antigen kit (ICkit) that is simple for all users can be used for point-of-care testing (POCT), is a standalone test without requiring any equipment, and provides rapid results with a decision time of 15 min was developed. Figure 1 shows the structure and principle of the ICkit. The ICkit targets the ribosomal protein L7/L12 antigen, which is a component of the 50S ribosome and contains an amino acid sequence specific to each bacterial species^20–23^. Sano et al. and Ito et al. reported the ICkit’s utility in detecting the ribosomal protein L7/L12 in diagnosing *Mycoplasma* pneumonia and *Legionella* pneumonia^24,25^. Similarly, the ICkit’s usefulness for detecting *B. pertussis* is confirmed in the present clinical study.

## Results

### Dilution sensitivity test

The results of the dilution sensitivity test are summarized in Table 1. The ICkit was positive with 2.2 × 10^4^ CFU/mL and more for all strains (BAA-589, 8467, 9797, and 9340). Therefore, the detection limit of the ICkit was estimated to be 2.2 × 10^4^ CFU/mL.

**Table 1:** Dilution sensitivity test with the ICkit for four different strains of *Bordetella pertussis*.

Strain (ATCC#)	Concentration (CFU/mL)	ICkit result
BAA-589	44 × 10^4^	+
	8.8 × 10^4^	+
	2.2 × 10^4^	+
	0.4 × 10^4^	−
8467	44 × 10^4^	+
	8.8 × 10^4^	+
	2.2 × 10^4^	+
	0.4 × 10^4^	−
9797	44 × 10^4^	+
	8.8 × 10^4^	+
	2.2 × 10^4^	+
	0.4 × 10^4^	−
9340	44 × 10^4^	+
	8.8 × 10^4^	+
	2.2 × 10^4^	+
	0.4 × 10^4^	−

*ICkit* immunochromatographic antigen kit, *+* ICkit-positive, − ICkit-negative.

### Cross-reactivity test

The cross-reactivity test identified cross-reactivity of the ICkit with *B. parapertussis* and *B. holmesii*. However, it identified no cross-reactivity with pathogens other than *B. parapertussis* and *B. holmesii* in the ICkit (see Supplementary Table S1 online).

### Clinical study

Between October 2016 and September 2019, a total of 203 patients gave consent and were enrolled in this study. Finally, 195 cases were analyzed for clinical evaluation of the ICkit. Eight cases were excluded from the analysis set because it was found after the study that they did not meet the inclusion criteria or did not have any ICkit results.

There were 68 infants, 83 children, 10 adolescents, and 34 adults in this study. The median age was three years (range 0–69 years). Their baseline demographics are summarized in Table 2, and their clinical symptoms are summarized in Table 3. The two tables are displayed in two groups: 14 years old or younger (for pediatric cases) versus 15 years old or older (for adolescent and adult cases). At baseline, the group aged 14 years or younger had a higher proportion of males, inpatients, vaccination, and antimicrobials before testing than the group aged 15 years or older. This seemed to be due to the fact that many adolescents and adults had milder symptoms and unknown vaccination status. Both groups had contacts with infected cases at a rate of about 30%. Clinical symptoms were similar to those previously reported^1^. All cases except one had a cough, whereas 54 (27.7%) of 195 cases had a fever. The median duration of cough up to the time of testing was 13 days (range 2–149 days). Whooping, paroxysms, post-tussive vomiting, and apnea are the characteristic symptoms of pertussis^1,26^. Whooping and apnea were found mostly in the group aged 14 years and younger. Both groups had a similar rate of paroxysms and post-tussive vomiting.

**Table 2:** Baseline demographics.

			14 years or younger (151 cases)		15 years or older (44 cases)	
**Sex**						
Male			85	56.3%	11	25.0%
Female			66	43.7%	33	75.0%
**Severity**						
Outpatient			96	63.6%	43	97.7%
Inpatient			55	36.4%	1	2.3%
**Vaccination**						
Present		1 time	9	6.0%	0	0.0%
		2 times	2	1.3%	0	0.0%
		3 times	14	9.3%	2	4.5%
		4 times	57	37.7%	9	20.5%
		Unknown^a^	10	6.6%	2	4.5%
Absent			49	32.5%	0	0.0%
Unknown			10	6.6%	31	70.5%
**Antimicrobials before testing**						
Present^b^			46	30.5%	7	15.9%
Absent			104	68.9%	34	77.3%
Unknown			1	0.6%	3	6.8%
**Contact with infected cases**						
Present			57	37.7%	15	34.1%
Absent			94	62.3%	29	65.9%

A total of 195 cases were analyzed. Of them, 151 cases were 14 years or younger, and 44 cases were 15 years or older.

^a^Vaccination is present, but the number of vaccinations is unknown.

^b^Macrolides were administered in 21 cases, cephem antibiotics in 18 patients, penicillin in 7 patients, and other antimicrobials in 7 patients.

**Table 3:** Clinical symptoms.

	14 years or younger (151 cases)		15 years or older (44 cases)	
**Fever**				
Positive	49	32.5%	5	11.4%
Negative	102	67.5%	39	88.6%
**Cough**				
Positive	150	99.3%	44	100.0%
Negative	1	0.7%	0	0.0%
**Duration of cough (days)**				
< 15	79	52.3%	22	50.0%
15 to 28	45	29.8%	15	34.1%
> 28	26	17.2%	7	15.9%
None	1	0.7%	0	0.0%
**Paroxysms**				
Positive	119	78.8%	36	81.8%
Negative	32	21.2%	8	18.2%
**Whooping**				
Positive	27	17.9%	2	4.5%
Negative	124	82.1%	42	95.5%
**Post-tussive vomiting**				
Positive	57	37.7%	15	34.1%
Negative	94	62.3%	29	65.9%
**Apneas**				
Positive	31	20.5%	0	0.0%
Negative	120	79.5%	44	100.0%

The correlations of the ICkit and culture results with real-time PCR (rPCR) results are summarized in Table 4. The sensitivity and the specificity of the ICkit were 86.4% (19/22) and 97.1% (168/173) compared with rPCR, and those of culture were 63.6% (14/22) and 97.6% (160/164) compared with rPCR, respectively. The sensitivity and the specificity of the ICkit were 72.2% (13/18) and 94.0% (158/168), respectively, compared with the culture (see Supplementary Table S2 online).

**Table 4:** Sensitivity, specificity, and concordance rate of the ICkit and culture compared with rPCR.

		ICkit			Culture		
		Positive	Negative	Total	Positive	Negative	Total
**rPCR**	Positive	19	3	22	14	8	22
	Negative	5	168	173	4	160	164
	Total	24	171	195	18	168	186
Sensitivity		86.4%			63.6%		
95% CI of sensitivity		66.7–95.3%			43.0–80.3%		
Specificity		97.1%			97.6%		
95% CI of specificity		93.4–98.8%			93.9–99.0%		

A total of 195 cases were analyzed. Nine cases were tested by the ICkit and rPCR, but not tested by culture.

*ICkit* immunochromatographic antigen kit. *rPCR* real-time polymerase chain reaction. *95% CI* 95% confidence interval.

**Figure 2:**
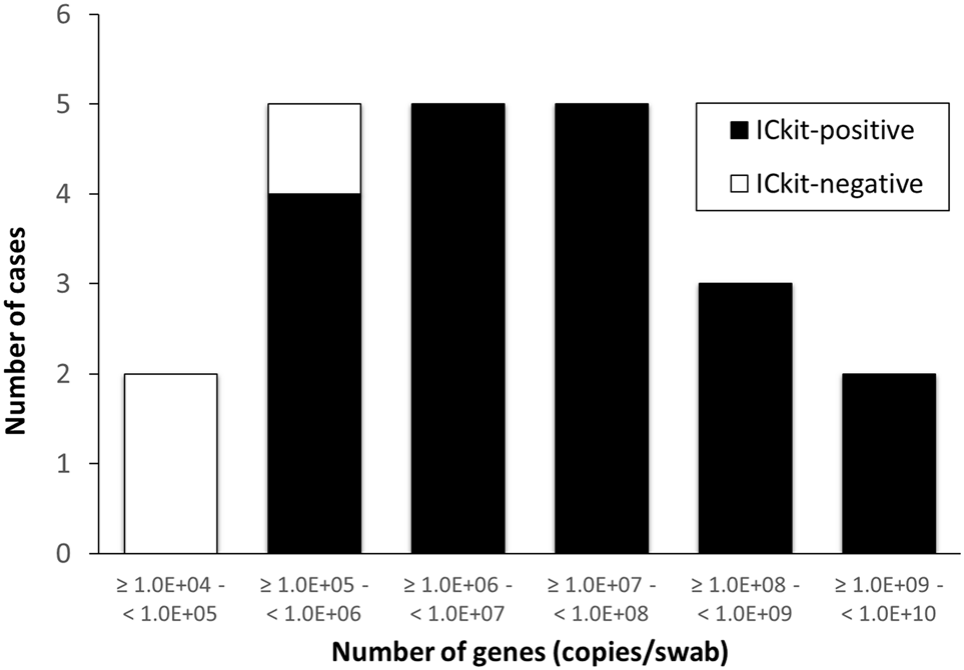
The relationship between the results of the ICkit and the number of *IS481* genes by rPCR. A total of 22 positive cases were analyzed by rPCR. In the three negative cases, *IS481* genes were detected by rPCR, but the ICkit was negative. *ICkit* immunochromatographic antigen kit, *rPCR* real-time polymerase chain reaction.

The relationship between the results of the ICkit and the number of *IS481* genes is shown in Fig. 2. There were many positive cases on rPCR from 1.0 × 10^5^ to 1.0 × 10^7^ copies/swab. The ICkit was negative in order from the group with the fewest copies in a swab. The ICkit was negative in two cases with 1.0 × 10^4^ copies/swab and in one case with 1.0 × 10^5^ copies/swab. Therefore, the detection limit of the ICkit was estimated to be around 1.0 × 10^5^ copies/swab.

**Figure 3:**
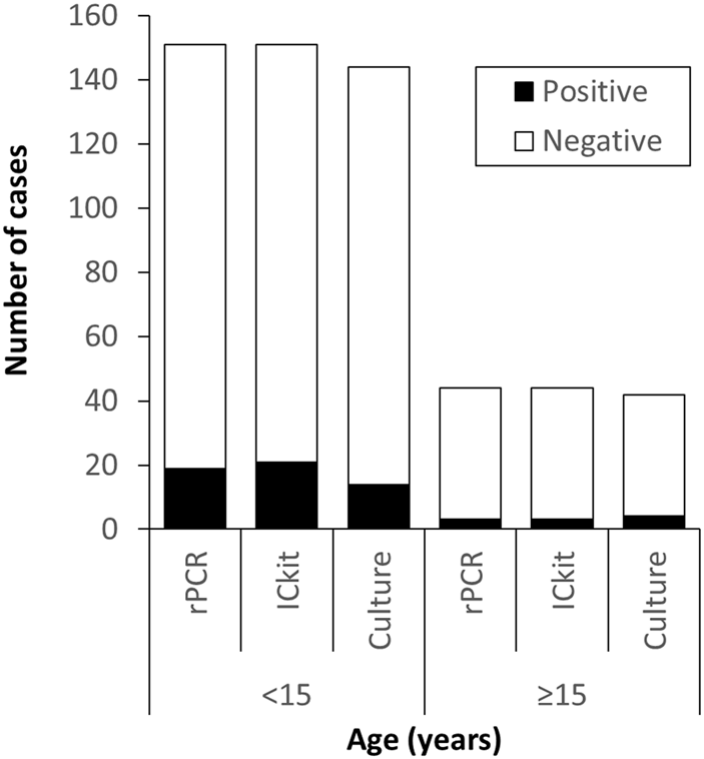
The results of rPCR, the ICkit, and culture by age group. A total of 195 cases were analyzed. Nine cases were tested by the ICkit and rPCR, but not tested by culture. *ICkit* immunochromatographic antigen kit, *rPCR* real-time polymerase chain reaction.

**Figure 4:**
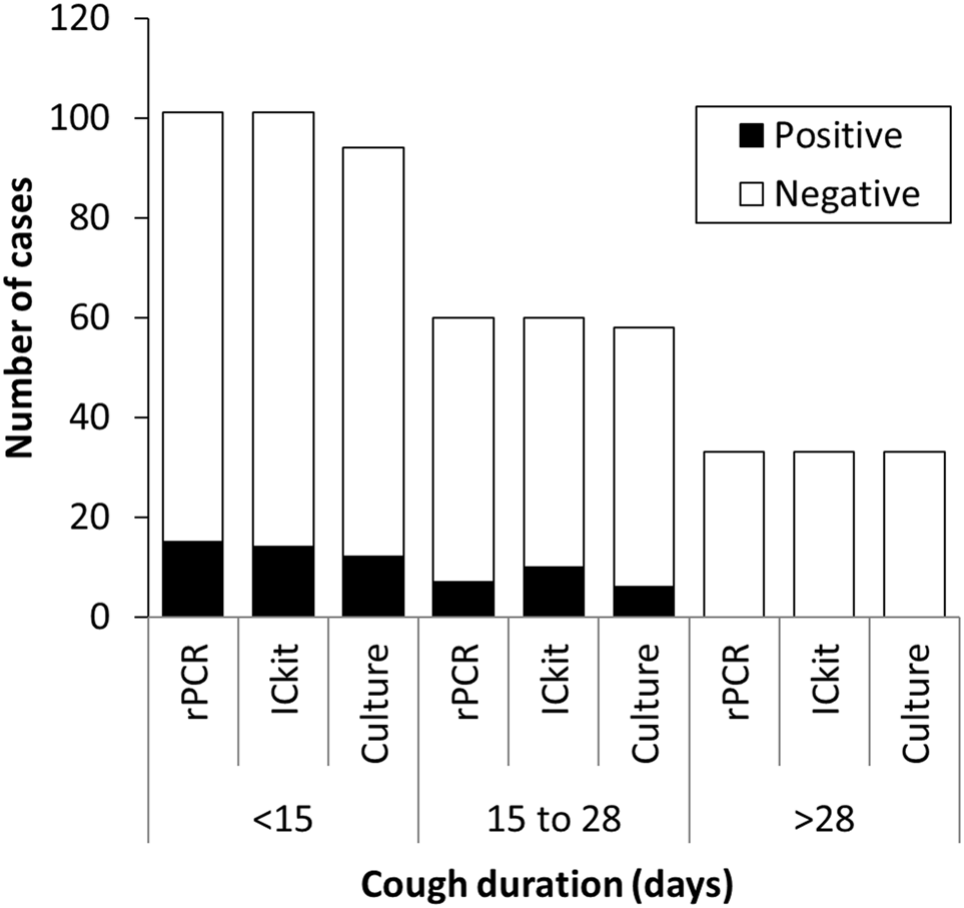
The results of rPCR, the ICkit, and culture by cough duration groups. A total of 194 cases with cough were analyzed. Of them, nine cases were tested by the ICkit and rPCR, but not tested by culture. *ICkit* immunochromatographic antigen kit, *rPCR* real-time polymerase chain reaction.

Both groups aged 14 years and younger and 15 years or older had positive cases with the ICkit, similar to rPCR (Fig. 3). However, each test’s proportion of positive cases was higher in the group aged 14 years and younger than in those aged 15 years or older. There were three cases that were ICkit-positive in the group aged 15 years or older. Of the three positive cases, two were positive on both rPCR and culture. Another was positive on rPCR and negative on culture and was not identified as *B. pertussis* or *B. holmesii*. The relationship between the test results and infection duration is summarized in Fig. 4. Infection duration was defined as the number of days from cough onset to testing. The number of positive cases with the ICkit was almost the same as the number of positive cases with rPCR for each duration. None of the tests had positive cases in the group longer than 28 days. The ICkit and rPCR provided positive results up to day 25, and culture provided positive results up to day 20.

## Discussion

*Pertussis* is a highly contagious infectious disease that is still widespread in both children and adults. The problem with pertussis is that unvaccinated infants are at risk of severe illness and death. Moreover, people around the infants can be the source of infection due to the decreased effectiveness of the vaccine. Nevertheless, no tests can detect the infection early and efficiently enough to treat or prevent the disease^12^. Therefore, a new POC device has been developed for rapid diagnosis of pertussis in recent years^27^. All existing tests require specialized equipment and trained personnel, which makes it difficult to access the tests. In addition, pertussis is highly infectious, so it is essential to have a test that provides rapid results at the patient’s first visit. We need to improve the inadequate access to testing for infected people. Especially in primary care facilities that most infected people visit first, it takes time to obtain test results due to the lack of testing capability.

We developed an immunochromatographic antigen test to improve access to testing. The ICkit result can be checked in 15 min after dispensing the sample onto the specimen loading area of the test plate without specialized equipment. The dilution sensitivity test confirmed that the ICkit could detect the antigens of *B. pertussis* strains. Furthermore, this was the first prospective clinical study using the ICkit for the diagnosis of pertussis. The usefulness of the ICkit was evaluated in 195 cases suspected of being infected with *B. pertussis*. Their baseline demographics and clinical symptoms (Tables 2 and 3) were similar to those previously reported^1^. There were many inpatient cases in infants without vaccination (see Supplementary Table S3 online), whereas there were many mild cases in adults. Antimicrobial treatment started before confirming the laboratory results due to its seriousness when inpatients and infants had probable pertussis. Several people were enrolled in the study after contact with people suspected of having pertussis. Almost all cases had a cough, but fewer cases with probable pertussis had a fever than with other infections. The duration of the cough was less than 28 days in most cases. For specific symptoms, paroxysms and post-tussive vomiting were common in children and adults. On the other hand, whooping and apnea episodes were seen in children, but rarely in adults.

The clinical performance of the ICkit was well correlated with its sensitivity of 86.4% (19/22) and specificity of 97.1% (168/173) compared to rPCR. Even in unvaccinated infants who are at risk of a serious disease course, the sensitivity and specificity of the ICkit were 87.5% (14/16) and 98.1% (51/52), respectively, compared with rPCR, similar to the other patients. The ICkit’s specificity (94.0%) compared to culture was high, but its sensitivity (72.2%) compared to culture was lower than that compared to rPCR (see Supplementary Table S2 online). When comparing the ICkit and culture to rPCR, the sensitivity of the ICkit was higher than that of culture (Table 4). Since rPCR is the most sensitive of pertussis tests^28^, the sensitivity of the ICkit, which correlated well with the sensitivity of rPCR, is considered to be higher than that of culture.

There were eight cases in which the results of rPCR and the ICkit did not match. In the three cases that were rPCR-positive and ICkit-negative, it was suspected that the results of the ICkit were false-negative due to an antigen amount below the detection limit of the ICkit, because the three cases had a smaller number of *IS481* genes in the swabs, as shown in Fig. 2. There is a similar report on the relationship between quantitative rPCR and culture^29^. This result showed that it is crucial to secure enough antigen by proper sample collection (i.e., correct method of sample collection or collecting sample at the early stage of infection) to obtain optimal results with the ICkit. For instance, the CDC describes a precise method for collecting a nasopharyngeal (NP) swab that applies to direct tests for *B. pertussis*^30^. The doctors’ diagnoses also supported positive results for pertussis in the three cases. In the five cases that were rPCR-negative and ICkit-positive, one of them seemed to be a false-negative rPCR result based on the doctor’s diagnosis. The other four cases were presumed to be false-positive results of the ICkit. False-positive results for immunochromatographic antigen kits have been reported to be caused by non-specific reactions of the kits when the specimen contains a large number of substances such as viscous substances and human anti-mouse antibodies (HAMAs)^31^.

Moreover, it was not possible to determine if the false-positive results were caused by cross-reactivity with *B. parapertussis* and *B. holmesii* in the present study (see Supplementary Table S1 online). This is because the L7/L12 protein sequences of *B. parapertussis* or *B. holmesii* are the same or almost the same as those of *B. pertussis*. Even if *B. parapertussis* or *B. holmesii* causes the infection, the treatment is the same as for *B. pertussis* infection. The doctor’s diagnosis also suggested false-positive results with the ICkit. Therefore, we must remember that the ICkit may show false-positive results if specimens contain viscous substances, HAMAs, *B. parapertussis*, or *B. holmesii*. The ICkit seems to be more specific in infants and children, since there are reports of less detection of *B. holmesii* in infants and children than in adolescents and adults^32–34^. When the ICkit result is suspected to be a false-positive result, we should consider confirming it with a PCR/culture-based test according to the global/national guidelines and differentiating it from cross-reaction with different *Bordetella* species.

In the present study, the ICkit detected the antigen in both children and adults similarly to rPCR (Fig. 3). However, the ICkit may produce false-negative results due to the disappearance of the bacteria if time has passed since onset or if patients take antimicrobials. Therefore, caution is needed regarding the timing of ICkit use. The CDC recommends the implementation of culture within two weeks and PCR within four weeks^10^. The period during which the ICkit detected the antigen was up to the 25th day, the same as rPCR in the clinical study (Fig. 4). Nakamura et al. reported that colonization tended to be less in adults than in children^35^. In addition, adults often have mild symptoms with atypical cough for pertussis and may delay seeking medical attention^36^. Therefore, we should ask for epidemiological information such as household infections and outbreaks to use the ICkit early after onset. In the present study, all ICkit-positive cases in adults were tested within two weeks of contact with infected cases. When the antigen has already disappeared over time, an antibody test should be an option for diagnosis^37,38^. Although the results of the present study were limited to three positive cases of rPCR in adults, further consideration of how to use the ICkit in adults is needed.

The characteristics of the ICkit’s rapidity and convenience suggest three major points. First, the ICkit would prevent infected people from getting more severe illness. The ICkit would contribute to early diagnosis and increase the opportunities for administering a macrolide, which is the first-line agent for pertussis, in the early stage^4,39^. If macrolides can suppress bacterial growth during the catarrhal phase, they can suppress the production of toxins that exacerbate respiratory symptoms. Based only on clinical symptoms, it is often difficult to distinguish *B. pertussis* infection from viral and other bacterial respiratory tract infections without any characteristic symptoms, which delays the administration of macrolides^1^. Both clinical symptoms and test results are important to identify and treat pertussis in the early stage. Second, the ICkit would help in antimicrobial stewardship^40,41^. In the present study, about 30% of cases received antimicrobials before laboratory confirmation. The shorter turnaround time with the ICkit enables doctors to check the test results on the spot and to decide whether patients need to receive antimicrobials within the first consultation. If the test results rule out pertussis and a viral respiratory tract infection is suspected, we will refrain from unnecessary antibiotics. Due to the side effect of hypertrophic pyloric stenosis, we must avoid unnecessary macrolides in infants without testing^42,43^. Antimicrobial stewardship is essential, because antimicrobial-resistant strains of *B. pertussis* have already been identified^44–46^. Third, the ICkit would contribute to preventing spread of the infection. Delay in diagnosis and treatment increases the risk of transmission and further expands large-scale outbreaks^11,47^. The ICkit will be useful in environments where culture and nucleic acid tests are not actively available or in urgent situations such as an outbreak. In such situations, the easy accessibility of the ICkit would be beneficial for checking whether people around the infected person are infected. In institutions that have difficulty performing tests, the ICkit would make it possible to identify *B. pertussis* that has been missed so far. The potential benefits of the ICkit would be maximized in low-resource countries with many infected people due to low vaccine coverage and where conventional testing for pertussis is difficult due to immature infrastructure^48,49^.

In conclusion, a new, rapid immunochromatographic antigen kit for *B. pertussis* was developed. Its procedure is simpler and faster than conventional tests, so that its result is available at the first visit of potentially infected people. Furthermore, the ICkit is a POCT that does not require any equipment and can be easily used in any clinical setting. The dilution sensitivity test and the clinical study demonstrated that the ICkit could detect the *B. pertussis* antigen. The clinical performance of the ICkit was well correlated with the rPCR results and was demonstrated within the same time from cough onset as rPCR. However, to correctly interpret the ICkit’s results, we must use the ICkit with a complete understanding of the ICkit’s precautions, such as cross-reactivity, the method for collecting the specimen, and the timing of use. Easy and rapid detection of *B. pertussis* by the ICkit would help diagnose pertussis more rapidly and efficiently.

## Methods

### Dilution sensitivity test

The dilution sensitivity test was conducted at Asahi Kasei Pharma. The test used four *Bordetella* strains, including BAA-589 (ATCC), 8467 (ATCC), 9797 (ATCC), and 9340 (ATCC). These strains were grown in *Bordetella* CFDN agar plates (Nikken Bio Medical Laboratory Inc., Kyoto, Japan) for 4 to 7 days at 37 °C to a concentration of 1.0 × 10^9^ colony forming units/mL (CFU/mL). These cultured strains were then suspended in phosphate-buffered saline (PBS) and used to investigate the detection limit of the ICkit. Samples applied to the ICkit were prepared from bacterial suspensions, reagent A, and reagent B. The test was performed at concentrations of 0.4 × 10^4^, 2.2 × 10^4^, 8.8 × 10^4^, and 44 × 10^4^ CFU/mL, respectively. Experiments were performed three times per concentration.

### Clinical study

This prospective clinical study was conducted from October 2016 to September 2019 in 11 institutions in Japan. This study was approved by the ethics committees of Asahi Kasei Pharma, Tokyo Metropolitan Children’s Medical Center, Konan Kosei Hospital, Miyazaki Prefectural Miyazaki Hospital, National Hospital Organization Fukuoka National Hospital, National Center for Child Health and Development, Yokohama Minoru Clinic, Sapporo Medical Association, and Aichi Children’s Health and Medical Center. All procedures were performed in accordance with the Declaration of Helsinki and its amendments. All patients or acceptable representatives provided written, informed consent. The contents of the clinical study are published on UMIN-CTR (UMIN000041828). The subjects were patients suspected of being infected with *B. pertussis*. Patients who matched either of the following were enrolled and specimens taken: (1) patients who had a cough and presented with one or more characteristic symptoms of paroxysms, whooping, post-tussive vomiting, or apnea with or without cyanosis; or (2) patients who had a possibility of contact with an infected person. Patients from whom nasopharyngeal swab specimens were not available or who were judged by doctors to be inappropriate for this study were excluded. In every institution, two swab specimens were taken per patient by rubbing the posterior nasopharynx. One swab specimen was immediately used for the ICkit measurement at each institution. The residual specimen of the ICkit measurement was used for the rPCR measurement at Kawasaki Medical School. The other swab specimen was immediately used for culture at each institution. The ICkit, the rPCR, and culture were each performed only once.

### Immunochromatographic antigen kit (ICkit)

The ICkit was approved as Ribotest Pertussis (Asahi Kasei Pharma, Tokyo, Japan) in Japan for the purpose of detecting *B. pertussis* antigens in a nasopharyngeal swab specimen. Figure 1 shows the structure and principle of the ICkit. The procedure of the ICkit is performed by following the steps (see also Supplementary Fig. S1 online).

- Take a postnasal specimen with a swab.
- Elute the specimen into reagent A in a tube.
- Add reagent B into the tube and mix, with inversion of the extraction tube closed by a cap.
- Dispense the sample onto the specimen loading area of the test plate.
- Determine the result based on the presence or absence of a line in the determination part of the test plate in 15 min (see Supplementary Fig. S2 online).

The cross-reactivity test confirmed that the ICkit has cross-reactivity with *Bordetella parapertussis* and *Bordetella holmesii* (see Supplementary Table S1 online).

### Real-time polymerase chain reaction (rPCR)

rPCR targeting *IS481* was performed using the previously described method with some modifications^28^. In total, 50 µL of the diluted test sample were used for DNA extraction, and total DNA was extracted using the QIAamp DNA Micro Kit (Qiagen, Hilden, Germany). DNA amplification was performed using the CFX96 Real-Time System (Bio-Rad, Hercules, CA, USA) and SsoFast Probes Supermix (Bio-Rad). In this study, *IS481* plasmid DNA was used as the internal control. A standard curve was also generated with tenfold serial dilutions of *IS481* plasmid DNA from 1.0 × 10^5^ copies to 1.0 × 10^2^ copies.

### Culture

*B. pertussis* was cultivated following the usual procedure at each institution. The culture medium for *B. pertussis* was charcoal agar medium (Oxoid Ltd., Hampshire, UK) at Konan Kosei Hospital, cyclodextrin pyruvate solid medium at Miyazaki Hospital, and *Bordetella* CFDN agar medium (Nikken Bio Medical Laboratory Inc.) at the other institutions. Finally, *B. pertussis* was identified by an agglutination test.

### Statistical analysis

Statistical analysis was performed using Analyse-it version 5.66 (Analyse-it Software, Ltd., Leeds, UK). Sensitivity, specificity, and their 95% confidence intervals were calculated with Analyse-it.

## Supplementary Information

Supplementary Information.

## Data Availability

The datasets generated and/or analyzed during the current study are available from the corresponding author on reasonable request.
