# Supplementary material for: Clinical evaluation of a new rapid immunochromatographic test for detection of Bordetella pertussis antigen
Source: Sci Rep. 2022 May 16;12:8069. doi: 10.1038/s41598-022-11933-y (PMC9109659; doi:10.1038/s41598-022-11933-y)
Supplement: Supplementary file 1 — Supplementary Information. [file 41598_2022_11933_MOESM1_ESM.pdf]

Supplementary information

Clinical evaluation of a new rapid immunochromatographic test for detection of *Bordetella pertussis* antigen

Kenji Okada[1], Yuho Horikoshi[2], Naoko Nishimura[3], Shigeki Ishii[4], Hiroko Nogami[5], Chikako Motomura[6], Isao Miyairi[7], Naoki Tsumura[8], Toshihiko Mori[9], Kenta Ito[10], Shinichi Honma[11], Kensuke Nagai[12], Hiroshi Tanaka[13], Toru Hayakawa[14]*, Chiharu Abe[14] & Kazunobu Ouchi[15]

[1] Division of Basic Nursing, Fukuoka Nursing College, Fukuoka, Japan

[2] Division of Infectious Diseases, Department of Pediatrics, Tokyo Metropolitan Children's Medical Center, Tokyo, Japan

[3] Department of Pediatrics, Konan Kosei Hospital, Aichi, Japan

[4] Department of Pediatrics, Miyazaki Prefectural Miyazaki Hospital, Miyazaki, Japan

[5] Department of Respiratory Medicine, National Hospital Organization Fukuoka National Hospital, Fukuoka, Japan

[6] Department of Pediatrics, National Hospital Organization Fukuoka National Hospital, Fukuoka, Japan

[7] Division of Infectious Diseases, Department of Medical Subspecialties, National Center for Child Health and Development, Tokyo, Japan

[8] Tsumura Family Clinic, Fukuoka, Japan

[9] Department of Pediatrics, NTT East Sapporo Hospital, Hokkaido, Japan

[10] Department of General Pediatrics, Aichi Children's Health and Medical Center, Aichi,

Japan

[11] Honma Children's Clinic, Fukuoka, Japan

[12] Nagai Pediatric Clinic, Fukuoka, Japan

[13] Sapporo Cough Asthma and Allergy Center, Hokkaido, Japan

[14] Asahi Kasei Pharma Corporation, Tokyo, Japan

[15] Department of Medical Welfare for Children, Kawasaki University of Medical Welfare, Okayama, Japan

*Corresponding author:

Toru Hayakawa

Diagnostics Department, Asahi Kasei Pharma Corporation, Tokyo, Japan

1-1-2 Yurakucho, Chiyoda-ku, Tokyo 100-0006, Japan

Tel: +81-3-6699-3617

E-mail: hayakawa.th@om.asahi-kasei.co.jp

Supplementary Table S1. Cross-reactivity of the ICkit with general bacterial pathogens.

Bacterial strain	ICkit Result	Bacterial strain	ICkit Result
Bordetella parapertussis (ATCC® BAA-587)	+	*Propionibacterium acnes* (ATCC® 6919)	–
Bordetella holmesii (ATCC® 700053)	+	*Streptococcus mitis* (ATCC® 49456)	–
Streptococcus aureus subsp. *aureus* (ATCC® 25923)	–	*Streptococcus oralis* (NCTC 11427)	–
Streptococcus epidermidis (ATCC® 14990)	–	*Streptococcus intermedius* (NCDO 2227)	–
Bacillus subtilis subsp. *subtilis* (ATCC® 6051)	–	*Streptococcus anginosus* (NCTC 10713)	–
Moraxella catarrhalis (ATCC® 25240)	–	*Streptococcus pneumoniae* (ATCC® 27336)	–
Escherichia coli (ATCC® 25922)	–	*Enterococcus faecalis* (ATCC® 19433)	–
Serratia marcescens (ATCC® 13880)	–	*Neisseria meningitidis* (ATCC® 13090)	–
Klebsiella pneumoniae (ATCC® 13883)	–	*Neisseria lactamica* (ATCC® 23970)	–
Pseudomonas aeruginosa (ATCC® 27853)	–	*Neisseria gonorrhoeae* (ATCC® 43070)	–
Proteus mirabilis (ATCC® 12453)	–	*Haemophilus influenzae* (ATCC® 10211)	–
Streptococcus pyogenes (ATCC® 19615)	–	*Haemophilus parahaemolyticus* (ATCC® 10014)	–
Streptococcus agalactiae (ATCC® 12386)	–	*Haemophilus haemolyticus* (ATCC® 33390)	–
Streptococcus salivarius (ATCC® 13419)	–	*Haemophilus parainfluenzae* (ATCC® 33392)	–
Streptococcus mutans (ATCC® 25175)	–	*Mycoplasma pneumoniae* (ATCC® 15531)	–

The ICkit was used for each pathogen at a concentration of 1.25×10^6 CFU/mL.

ICkit: immunochromatographic antigen kit. +: ICkit-positive. −: ICkit-negative.

Supplementary Table S2. Sensitivity, specificity, and concordance rate of the ICkit compared with culture.

		ICkit		
		Positive	Negative	Total
Culture	Positive	13	5	18
	Negative	10	158	168
	Total	23	163	186
Sensitivity		72.2%		
95% CI of sensitivity		49.1% to 87.5%		
Specificity		94.0%		
95% CI of specificity		89.4% to 96.7%		

A total of 186 cases with results from both the ICkit and cultures were analyzed.

ICkit: immunochromatographic antigen kit. 95% CI: 95% confidence interval.

Supplementary Table S3. Baseline demographics of the infants under one year of age.

			Infants under one year of age (68 cases)	
Sex	Male		40	58.8%
	Female		28	41.2%
Severity	Outpatient		22	32.4%
	Inpatient		46	67.6%
Vaccination	Present	1 time	9	13.2%
		2 times	2	2.9%
		3 times	7	10.3%
		Unknown[a]	1	1.5%
	Absent		48	70.6%
	Unknown		1	1.5%
Antimicrobials before testing	Present[b]		29	42.6%
	Absent		39	57.4%
Contact with infected cases	Present		29	42.6%
	Absent		39	57.4%

A total of 195 cases were analyzed. Of them, 68 cases were infants under one year of age.

[a] Vaccination is present, but the number of vaccinations is unknown.

[b] Macrolides were administered in 12 cases, cephem antibiotics in 13 patients, penicillin in 3 patients, and other antimicrobials in one patient.

Supplementary Figure S1. Assay procedure of the ICkit.

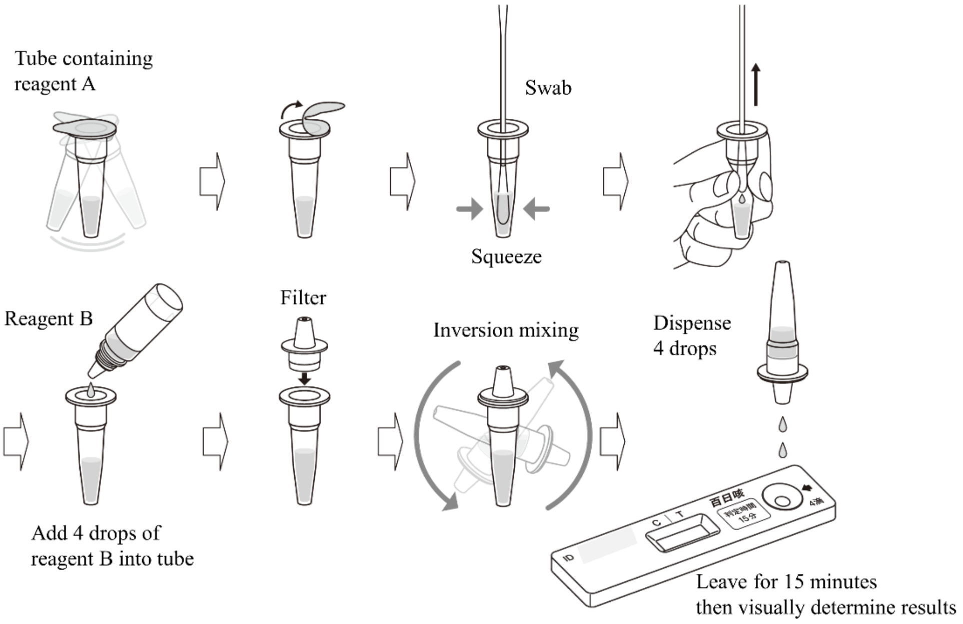

Supplementary Figure S2. Actual ICkits showing positive and negative results.

A

B

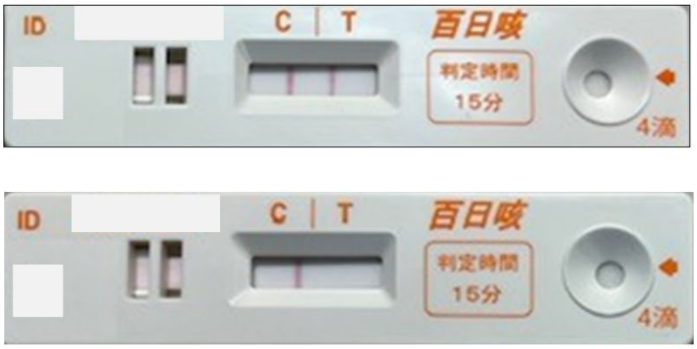

A: ICkit-positive. B: ICkit-negative.
